# START – physical exercise and person-centred cognitive skills training as treatment for adult ADHD: protocol for a randomized controlled trial

**DOI:** 10.1186/s12888-023-05181-1

**Published:** 2023-09-25

**Authors:** Mialinn Arvidsson Lindvall, Kajsa Lidström Holmqvist, Lena Axelsson Svedell, Anna Philipson, Yang Cao, Mussie Msghina

**Affiliations:** 1 University Health Care Research Centre, Faculty of Medicine and Health, Örebro University, Örebro, 70182 SE Sweden; 2 Department of Neurology and Rehabilitation Medicine, Faculty of Medicine and Health, Örebro University, Örebro, 70182 SE Sweden; 3 Department of Psychiatry, Faculty of Medicine and Health, Örebro University, Örebro, 70182 SE Sweden; 4 Clinical Epidemiology and Biostatistics, School of Medical Sciences, Faculty of Medicine and Health, Örebro University, Örebro, 70182 SE Sweden; 5 Unit of Integrative Epidemiology, Institute of Environmental Medicine, Karolinska Institutet, Stockholm, Sweden; 6 Department of Clinical Neuroscience, Karolinska Institute, Stockholm, Sweden

**Keywords:** ADHD, Cognitive support, Intervention, Physical activity, RCT

## Abstract

**Background:**

Core symptoms in attention deficit hyperactivity disorder (ADHD) are inattention, impulsivity and hyperactivity. Many individuals with this disorder also have a sedentary lifestyle, co-morbid mental illness such as depressive and anxiety disorders, and reduced quality of life. People with ADHD often have impaired executive function, which among other things may include difficulty in time management and structuring of everyday life. Pharmacological treatment is often the first-line option, but non-pharmacological treatment is also available and is used in clinical settings. In children and adolescents with ADHD, physical exercise is used as a non-pharmacological treatment. However, the evidence for the effectiveness of exercise in adults is sparse.

**Objective:**

To implement the START intervention (START = *Stöd i Aktivitet, Rörelse och Träning* [Support in activity, movement and exercise]) consisting of a 12-week, structured mixed exercise programme with or without a cognitive intervention, in adults with ADHD, and study whether it has an effect on core symptoms of ADHD as well as physical, cognitive, mental and everyday functioning compared with usual treatment. A secondary aim is to investigate the participants’ experiences of the intervention and its possible benefits, and to evaluate the cost-effectiveness of START compared with usual treatment.

**Methods:**

This is a randomized controlled trial planned to be conducted in 120 adults with ADHD, aged 18–65. The intervention will be given as an add-on to standard care. Participants will be randomized to three groups. Group 1 will be given a physiotherapist-led mixed exercise programme for 12 weeks. Group 2 will receive the same intervention as group 1 with the addition of occupational therapist-led cognitive skills training. Group 3 will be the control group who will receive standard care only. The primary outcome will be reduction of ADHD symptoms measured using the World Health Organization (WHO) Adult ADHD Self-Report Scale (ASRS-v1.1), Clinical Global Impression-Severity scale (CGI-S) and CGI-Improvement scale (CGI-I). The effect will be measured within 1 week after the end of the intervention and 6 and 12 months later.

**Discussion:**

Data collection began in March 2021. The final 12-month follow-up is anticipated to be completed by autumn 2024.

**Trial registration:**

ClinicalTrials.gov (Identifier: NCT05049239). Registered on 20 September 2021 (last verified: May 2021).

## Background

The prevalence of attention deficit hyperactivity disorder (ADHD) in adults is estimated to be 2.5–5% [1, 2]. The core symptoms of ADHD are inattention, impulsivity and hyperactivity [3]. People with ADHD may also have impaired executive function, which among other things includes difficulty in time management and structuring of everyday life. Adults with ADHD have been shown to have difficulty finding and maintaining gainful employment and experience a high degree of stress in managing everyday life [4], leading to fatigue and poor sleep quality and quantity [5–7]. Brain imaging studies have shown structural and functional abnormalities in key brain areas including the basal ganglia and the cerebellum, which are more accentuated in children and adolescents than in adults. Some of these functional abnormalities may be normalized by pharmacological treatment with stimulant medication [8, 9].

People with ADHD have a high prevalence of comorbidity with other mental disorders such as anxiety disorders and depression [10]. A major risk factor for secondary diseases is also the patients’ sedentary lifestyle, with little movement and exercise [5–7, 10, 11]. Moreover, people with ADHD have substantial problems with emotion regulation, which can negatively impact interpersonal relationships both at home and at work [4].

Currently, the first-line treatment option in adult ADHD is pharmacological treatment with central stimulants (CSs) although guidelines recommend multimodal treatment [12]. The response rate to pharmacological treatment is high; however, up to 30% of patients fail to respond to CS medication [13]. There are a number of non-pharmacological treatment options that are used in the clinical setting, but the evidence for these is often lacking [10, 11], and there is a need for well-designed randomized controlled trials (RCTs) to evaluate them.

Physical activity improves mood and sleep, boosts energy, supports healthy ageing and lowers the risk for all-cause mortality by reducing, among other things, risks for cardiovascular disease and type-2 diabetes [14]. In children with ADHD, physical exercise has been shown to improve problems of inattention, hyperactivity and impulsivity [15–21], but in adults with ADHD there is still a lack of knowledge. In general, moderate to vigorous exercising for at least 150 min a week greatly reduces the risk of cardiovascular disease, diabetes and premature death and has major effects on the mental state and illnesses such as depression and anxiety [15, 16, 22]. Whether such activity levels can also reduce ADHD symptoms in adults remains to be investigated.

It is also not known whether, and how, physical exercise can affect the brain’s structure and function in adults with ADHD [18]. Physical exercise is a way to gain increased experience of the body and can strengthen a person’s confidence in their own physical ability [7, 23]. This is related to body awareness, which is the experience of “living in the body” and forms the basis for self-confidence and the ability to take care of oneself and one’s needs. Research has shown that an increased experience of the body provides increased wellbeing [24]. However, whether physical exercise improves body awareness in adults with ADHD remains to be investigated. To our knowledge there are no studies that examine how body awareness is affected by physical exercise in adults with ADHD or whether there is a relationship between emotion regulation, self-efficacy and body awareness in adults with ADHD. There is moderately strong scientific evidence that physical exercise can affect sleep quality and fatigue, regardless of etiology, and that energy levels increase in individuals who are physically active [16, 25]. However, there are no studies examining whether physical exercise improves sleep quality and perceived fatigue in adults with ADHD.

In a pilot study ahead of this planned RCT, an intervention including a mixed exercise programme (MEP) was evaluated in 14 adult participants with ADHD [26]. Nine participants were randomized to the intervention group, and five to the treatment as usual (TAU) control group. Three participants dropped out shortly after inclusion, before receiving any intervention; the remaining eleven (approx. 80%) conducted the intervention/treatment according to protocol. Overall, participants were satisfied and reported no major difficulties in following the intervention. The results were highly consistent with trends in post-intervention changes for almost all outcome variables.

One challenge that was seen in the pilot study was that some participants had difficulty in organizing and making time for the 12-week intervention. To plan, accomplish and evaluate activities, executive functioning is fundamental, which is also the case when it comes to performing physical activities. There are few intervention studies that have evaluated the effect of cognitive interventions for adults with ADHD; however, the few studies available show that cognitive skills training improves the everyday functioning of adults with ADHD [27–30]. These intervention studies regarding structured cognitive interventions including skills training for adults with ADHD report that implementation of person-centred strategies for planning daily activities improved the individuals’ performance and increased their self-esteem and confidence in their own ability [27, 29].

To conclude, there are currently several knowledge gaps in the treatment of adult ADHD. The evidence for physical exercise, or physical exercise in combination with a person-centred, structured cognitive intervention, as a treatment for ADHD symptoms in adults is deficient. There is therefore a great need for further studies to investigate whether physical exercise with or without cognitive intervention has the same effect on ADHD symptoms in adults as in children and adolescents [4, 17, 18, 20].

### Rationale

Regular physical activity has a positive effect on ADHD core symptoms in children and adolescents, but corresponding evidence regarding this effect on symptoms in adults is lacking and there is a great need to study this [2, 5, 14]. People with ADHD face challenges in their everyday life, challenges of inattention, impulsivity and hyperactivity, but they also exercise less and have a sedentary lifestyle. People with ADHD also struggle with the structure of everyday life and there are studies that show that structured cognitive skills training improves the everyday functioning of adults with ADHD. However, it has not been studied whether such training can contribute to increased physical activity or to maintaining routines for this. Today, the guidelines recommend multimodal treatment for people with ADHD, in other words, a combination of pharmacological and non-pharmacological interventions [12]. If our study can prove that the START intervention (START = *Stöd i Aktivitet, Rörelse och Träning* [Support in activity, movement and exercise]) can improve the core symptoms and quality of life (QoL) for adult patients with ADHD, it could be a welcome addition to the available treatment options. The goal is to develop a multimodal, non-pharmacological intervention of physical exercise with or without cognitive skills training as treatment for ADHD and to test this in an RCT.

### Aim

The main aim is to implement the 12-week START intervention consisting of a structured MEP with or without cognitive skills training in adults with ADHD, and to study whether it has an effect on core symptoms of ADHD as well as physical, cognitive, mental and everyday functioning, compared with treatment as usual (TAU). TAU consists of pharmacological or non-pharmacological treatment without physical exercise. A further aim is to describe the participants’ experiences of the intervention and its possible benefits, and to evaluate the cost-effectiveness of START compared with TAU.

## Method

### Design

This study is a planned prospective RCT in three parallel groups of adults diagnosed with ADHD, following the SPIRIT guidelines [31].

**Fig. 1:**
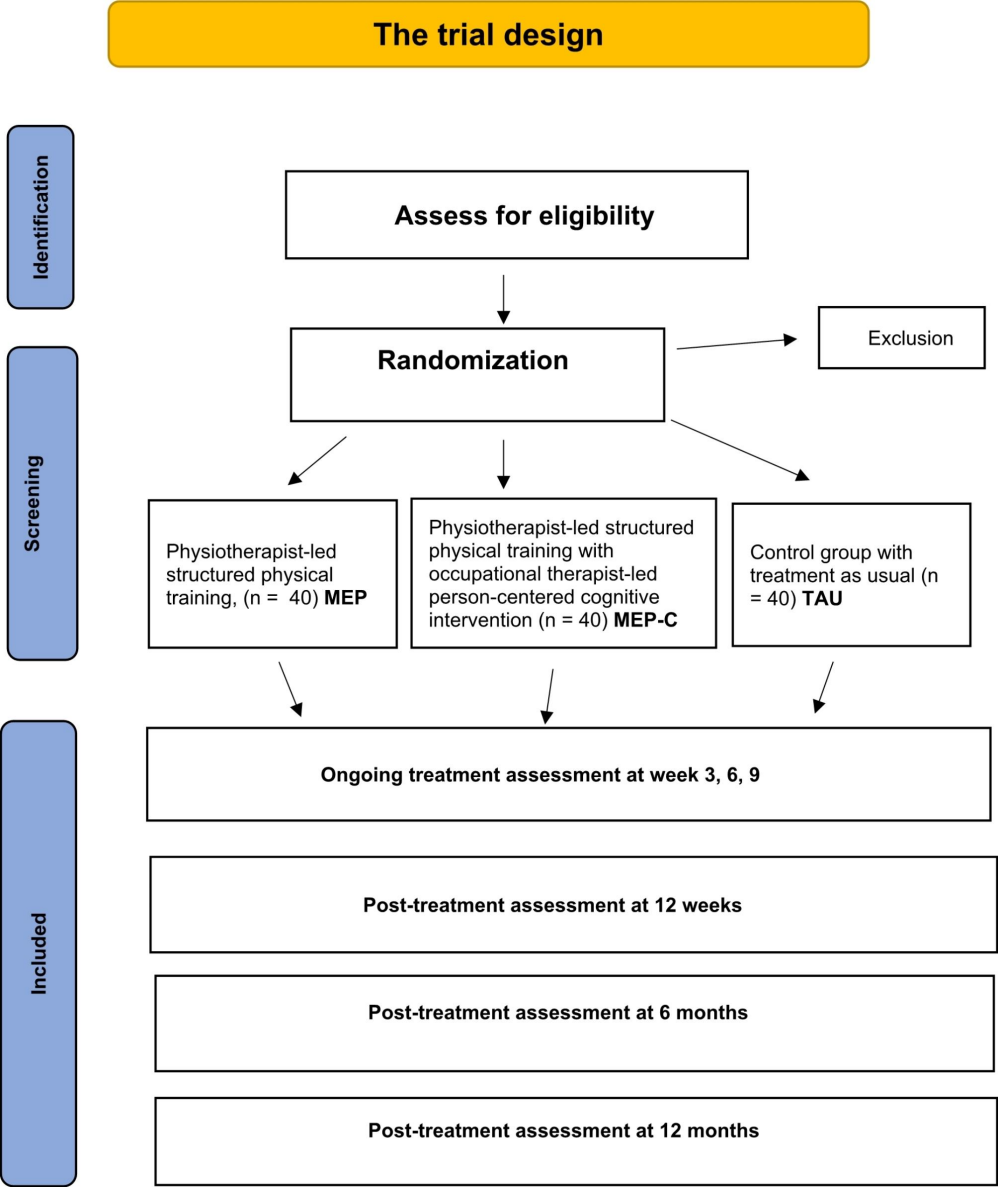
Flow diagram of trial design. MEP = a structured physiotherapy-led exercise programme; MEP-C – MEP together with an occupational therapist-led, person-centred cognitive intervention; TAU = treatment as usual

Randomization into three groups will be as follows:

1. Physiotherapist-led structured physical exercise group (n = 40);
2. Physiotherapist-led structured physical exercise group with occupational therapist-led, person-centred cognitive skills training (n = 40);
3. Control group receiving TAU (n = 40). For a flow chart of the trial design, see Fig. 1.

### Sample size estimation

The power calculation is based on the primary outcome, ADHD symptom reduction assessed using the World Health Organization (WHO) Adult ADHD Self-Report Scale (ASRS-v1.1) [32]. Postulating a mean difference in symptom reduction of 15% between the intervention and control groups, with 10.63% SD, 0.05 alpha, 0.8 power and expecting a 25% drop-out, gives a sample size of 120 participants. The actual sample size required for a power of 0.8 is 90 (= 120–120*25%). In case we have larger than 25% drop-out, we will recruit more participants to maintain the necessary power of 0.8.

### Participants

Recruitment will primarily be conducted by the General Psychiatry outpatient clinic and the clinic for young adults at Örebro University Hospital, Örebro, Sweden. Eligible persons will receive written and oral information about the study and a consent form when visiting the clinic. Participants will also be recruited via the following channels: the 1177 website (the official website for all county-based health care in Sweden); the Örebro County Facebook account; and the National Association of Neurodevelopmental Disorders, Riksförbundet Attention. Interested persons will be invited to contact the research team to receive written and oral information about the study and a consent form. Individuals who agree to participate in the study and are eligible according to the inclusion and exclusion criteria will be included. To assess eligibility for inclusion, their medical history will be retrieved from the medical records.

Inclusion criteria: Diagnosed with ADHD; age 18–65 years; stable medication or no medication.

Exclusion criteria: Ongoing depression (Montgomery-Asberg Depression Rating Scale, Self-Assessment (MADRS-S) score > 21), suicidality, bipolar disorder, psychosis, substance abuse, severe autism, reported threatening behaviour, and severe physical illness, as well as inability to read/understand Swedish.

### Randomization and blinding

Individuals meeting the inclusion criteria will be randomized into three groups (two intervention groups and one control group) using the Smart-Trial (S-T) electronic data capture platform hosted by Region Örebro County. Randomization will be performed in a 1:1:1 ratio using a block of 6 (9 or 12 to be determined). Stratification will not be applied. During the clinician-rated assessment interview, participants will be instructed not to disclose their group assignment. Due to the nature of the intervention involving structured physical exercise with or without additional person-centred cognitive skills training, blinding of participants to the interventionists in the planned RCT will not be feasible. The individuals collecting the physical tests will also not be blinded to group allocation. However, the data analysts will be blinded to group allocation during the analysis process.

### Intervention: the START intervention programme

The intervention consists of a MEP with and without cognitive skills training, which includes strength, cardio and flexibility training. One arm of the study will receive physical exercise together with cognitive skills training to address impaired executive function in everyday life, which is common among people with ADHD. This training is an occupational therapist-led, person-centred cognitive intervention aiming to improve time management skills, planning and organization. The physical exercise will consist of interval training, following a structured programme including both aerobic/endurance exercises and resistance exercises/strength training (see Table 1). The occupational therapy intervention will depend on the participant’s personal goals (Table 2).

**Table 1:** Structure of the interval training of the START intervention

START intervention exercise (total 45 min)		
Type/purpose	Duration (% of total)	Content
**Warm-up**	6 min (13.5%)	Light exercise, cycling, cross-training, walking or full body movements. “Focus on how you are today.”
**Cardio**	6 min (13.5%) (3 × 1-min exercise intervals, 1 min active rest between each interval)	Cycling, running/fast walking or jumping
**Resistance training**	23 min (51%) (1 min exercise + 20 s rest x 5 exercises in 3 rounds. 1 min rest between each round.)	Exercises for different muscle groups: lower body, upper body, core and balance. All groups are represented in each round, but exercises may vary. Number of repetitions are controlled by time limits and can vary with weight.
**Cool-down and flexibility**	10 min (22%)	Cool-down with light exercise, cycling, cross- training, walking or full body movements. Stretching exercises for larger muscle groups used in the resistance training. “Focus on how the body feels.”

**Table 2:** Occupational therapy intervention

Session	Contents
1	Assessment of occupational performance using the Canadian Occupational Performance Measure (COPM): includes ranking and prioritization of a maximum of five activity-based intervention goals. The goals are based on the participant’s estimation of level of satisfaction with the performance, and value in daily life. Assessment of Satisfaction with Daily Occupations-Occupational Balance (SDO-OB). The participant and the occupational therapist agree on the goal(s) that should be focused in the intervention.
2 to approx. 5	Intervention period. The number of sessions is based on the occupational therapist’s judgement of need depending on the goals set. Normally one session is held every second week. The intervention is focused on cognitive skills training using strategies targeting the activity limitations of the participant. This may include, but is not limited to: use of compensatory aids for planning and organization such as calendar, alarms and time visualization aids. The aim is for the participant to learn how to prioritize and estimate time, and build strategies to reach occupational balance in daily life (meaning balance between activity, recovery and sleep). For each session, the participant has home assignments, such as to try a new strategy or to keep a time diary to gain awareness of occupational patterns and occupational balance.
6	Evaluation of goal attainment using the COPM. Assessment using the SDO-OB.

1. **Type of intervention: MEP** – a structured physiotherapy-led exercise programme; or **MEP-C** – MEP together with an occupational therapist-led, person-centered cognitive intervention.
2. **Dose, MEP**: Physical exercise for 45 min, three times a week for 12 weeks.
   **Dose, MEP-C:** Occupational therapy for 60 min approximately six times (every second week) during the 12-week period depending on participant’s goals related to the intervention; and physical exercise for 45 min, three times a week for 12 weeks.
3. **Dose, MEP**: Physical exercise for 45 min, three times a week for 12 weeks.
   **Components of physical exercises, MEP and MEP-C**: All exercise sessions will have the same structure and consist of endurance, strength and flexibility training. Desired heart rate (HR) range will be 60–90% of maximum HR. In order to track HR during all sessions, both in the clinic and at home, the participants will have a Polar Heart Rate Monitor, model H10 (Polar Electro Oy, Kempele, Finland), connected to their mobile phone.
   Each exercise session will start with 5–10 minutes’ **warm-up** on an ergometer or using body movements. Next will be **endurance exercises** in three to five intervals of 1 min work and 1 min active rest between each interval. The **strength training exercises** will consist of five different exercises with 1-minute intervals between exercises, in three rounds. These strength exercises may vary depending on the location, indoors or outdoors. However, they will always include one exercise mainly for leg strength, for example squats or lunges; one exercise for arms and shoulders, for example shoulder press with barbell or bench press; one core exercise, for example crunches or the plank; and one balance challenge, for example standing on one leg. The participants will be encouraged to do as many repetitions as possible in 1 min and therefore the load of the exercise can be altered depending on the number of repetitions the participant can manage. In the cool-down, in the warm-up and during stretching the participant will be encouraged to sense the feeling of the body and their overall feeling.
4. **Components of the cognitive intervention of the MEP-C**: As previously mentioned, the cognitive intervention will be conducted by an occupational therapist on approximately six occasions during the 12-week period. Based on self-prioritized goals concerning structure, planning and organization in everyday life, a treatment plan for cognitive skills training will be drawn up. This will form the basis of the treatment. (Examples of such goals are: to arrive on time for training, or to create the space for physical activity in one’s everyday life through improved time management.)
5. **Sequences**: The training sessions will take place either in the training facilities at the outpatient clinic or outdoors in a nearby park. Each session will follow a specific structure.
6. **Modifications**: Alternative exercises will be suggested by the instructors when needed. Participants will be allowed to perform to the best of their ability, with encouragement from the physiotherapists to use maximum effort.
7. **Selection of instructors**: The instructors for the physical exercise sessions will be licensed physiotherapists at the Unit for Psychiatric Physiotherapy, Region Örebro County, who are experienced in working with persons with ADHD. The instructors for the cognitive intervention will be occupational therapists with experience of both working with persons with ADHD and using the Canadian Occupational Performance Measure (COPM) as person-centred tool [33, 34].
8. **Home practice**: In addition to the physiotherapist-led group, the participants will be expected to exercise individually once a week, to the extent needed to reach 150 minutes’ exercise per week. Participants receiving cognitive skills training will be given home assignments, primarily to implement compensatory cognitive strategies in daily life.
9. **Fidelity**: Training adherence will be noted by the treating physiotherapist and occupational therapist. Group adherence to physical activity will be calculated as the number of patients attending 50% of the sessions. To maintain physical exercise for at least 150 min per week, the HR monitor connected to the participants’ mobile phone will gather the time in an HR zone of > 60%. Attendance rate over the intervention period will be calculated as a mean of the individual percentage.
10. **Control group**: Participants randomized to the control group will continue with their TAU. This will include any ongoing pharmacological and/or non-pharmacological treatment that does not include physical exercise or cognitive skills training during the 12-week period.
   After the 12-week data collection, the participants will be offered physiotherapy-supported training outside the framework of the study.

### Data collection

Data will be collected before the intervention, during the intervention at week 3, 6 and 9, and directly after the 12-week intervention. Follow-up assessments will be at 6 and 12 months after the start of the intervention. One month after completion of the intervention, a consecutive sample from each of the two intervention groups (n = 15) will be interviewed regarding their experiences of the intervention and eventual benefits.

All online self-assessment scales will be completed digitally in the S-T electronic data capture platform. The data system will automatically send e-mails to participants informing them to log on to the portal to complete the questionnaires, and will notify them when an assessment is approaching or if a questionnaire has not been completed. Notifications will also be sent by e-mail if questionnaires are not completed within the set time frame. Participants who prefer paper questionnaires will have them sent by post together with an envelope with prepaid postage, and questionnaires will be stored in a locked facility at the University Health Care Research Centre at Örebro, to which only the research team will have access. Physical examinations and magnetic resonance imaging (MRI) examinations will be performed during visits to the outpatient clinic and X-ray clinic, respectively.

Data will be collected both in paper and digital formats and stored in locked fireproof cabinets when not being processed, or on a password-protected server within Region Örebro County (RÖL). The material collected will be provided with a code in place of personal data, and only authorized persons will have access to the code key and materials.

Only authorized project members will have access to all folders and subfolders. The principal investigator (MM) and data handlers will have authorization to read and write in the files under project documents, original data, and personal codes. Collected data will be stored in accordance with current legislation and local guidelines for at least 10 years after publication. All project data will be stored on the server, which can only be accessed at RÖL or via a virtual private network (VPN) or, outside RÖL’s area, with a password to the computer and VPN.

### Outcome measures

See Table 3 for a summary of measures given by time point.

**Table 3:** Measures by time point

	Completed by:	Visit 1	3 weeks	6 weeks	9 weeks	12 weeks (end of the intervention)	6- month follow- up	12- month follow- up
Informed consent to trial participation	PT							
**Primary outcome measures (ADHD symptoms)**								
MADRS-S*	PT					P	P	P
WHO Adult ADHD Self-Report Scale (ASRS-v1.1)		P		P		P	P	P
Clinical Global Impression-Severity (CGI-S)	AP					AP	AP	AP
Clinical Global Impression- Improvement (CGI-I)						AP	AP	AP
**Secondary outcome measures**								
Patient-rated Global Impression-Improvement (PGI-I)				p		P	p	p
Quality of life (QoL) assessed with the EQ-5D-5 L*		p		p	P	P	p	p
Ekblom-Bak (EB) submaximal exercise test		PT				PT	PT	PT
Balance on one leg (60-second Flamingo balance test)		PT				PT	PT	PT
Grip strength		PT				PT	PT	PT
Body mass index (BMI)		PT				PT	PT	PT
Abdominal circumference		PT				PT	PT	PT
Body Awareness Scale -Movement Quality and Experience (BAS MQ-E)		PT				PT	PT	PT
Physical activity and steps measured using an accelerometer, 7 days		P				P	P	P
Swedish National Board of Health and Welfare’s indicator questions about exercise and training habits		P	P	P	P	P	P	P
**Mental function**								
Cognitive tests on computer (AX-CPT*, Go/NoGo, IAPS*)		AP				AP		
Magnetic resonance imaging (MRI) examination		AP				AP		
**Everyday function**								
The Canadian Occupational Performance Measure (COPM)		OT				OT	OT	OT
Assessment of Time Management Skills, Swedish version (ATMS-S)		P				P	P	P
Satisfaction with Daily Occupations-Occupational Balance (SDO-OB)		OT				OT	OT	OT
Adult ADHD Quality of Life Questionnaire (AAQol)		P				P	P	P
General Self-Efficacy 10-item Scale (GSES-10)		P		P		P	P	P
Self-efficacy for exercise (SEE-S)		P	P	P	P	P	P	P
Insomnia Severity Index (ISI)		P	P	P	P	P	P	P
Interviews						OT, PT		

ADHD = attention deficit hyperactivity disorder. AP = assistant psychologist; OT = occupational therapist; P = participant; PT = physiotherapist.

*For the names of instruments not appearing in full here, please see the Abbreviations list

### Primary outcome measures

Changes in ADHD symptoms:

- The WHO Adult ADHD Self-Report Scale (ASRS-v1.1) [32]. The ASRS-v1.1 is a self-assessment form consisting of 18 questions corresponding to the 18 criteria for ADHD according to the Diagnostic and Statistical Manual of Mental Disorders, Text Revision, 4th edition (DSM-IV-TR). Part A consists of six questions that predict ADHD while part B consists of twelve questions about other symptoms. The ASRS-v1.1 has shown good reliability and validity [32].
- The Clinical Global Impression-Severity scale (CGI-S). The CGI-S is used for clinical assessment of the severity of symptoms [35]. The CGI–S scale reflects the patient’s total psychiatric impairment. It provides an overall clinician-determined summary measure that takes into account all available information, including knowledge of the person’s history, psychosocial circumstances, symptoms, and behaviour, and the impact of the symptoms on the person’s ability to function.
- The Clinical Global Impression-Improvement scale (CGI-I) [35]. This is a clinician-rated instrument which provides self-rated global measures of improvement and treatment satisfaction on a 7-point scale, from 1 = very much better to 7 = very much worse [36].

### Secondary outcome measures

Clinical variables:

- The Patient-rated Global Impression-Improvement scale (PGI-I) [35].
- The EQ-5D-5 L [37, 38], developed by the EuroQol Group. The EQ-5D-5 L is a QoL measure for assessing an individual’s health status, using a 5-level scale ranging from “no problem” to “extreme problems”, divided into five dimensions of mobility, self-care, usual activities, pain/discomfort, and anxiety/depression. The EQ-5D-5 L can also be used to generate a utility value that ranges from 0 (dead) to 1 (perfect health). By multiplying the time spent in a given health state (baseline – 3 weeks – 6 weeks – 9 weeks – 6 months – 12 months) by the average utility weight associated with the period (using the trapezium rule), quality-adjusted life years (QALYs) will be derived. This will be used in the health economic evaluation.

Physical function:

- The Ekblom-Bak (EB) submaximal exercise test. The EB test is a valid measure of maximal oxygen uptake (VO_2_max) for a wide variety of ages [39, 40].
- The Flamingo balance test is a 60-second one-leg stand test measuring static balance and stability in the abdominal, pelvic and leg muscles [41, 42].
- Grip strength measured with a hand-held dynamometer JAMAR, (Item 3363, G.E. Miller, Inc., 484 Broad- way, Yonkers, New York, 10,705) a reliable and valid measure in healthy participants as well as across various clinical populations [43, 44], will be tested, and height, weight and waist circumference will be measured using standardized tests [45]. Thereafter, body mass index (BMI) will be calculated.
- The Body Awareness Scale-Movement Quality and Experience (BAS MQ-E) will be used for physiotherapeutic assessment of body awareness [46]. The psychometric properties of this scale have been reported to be good [47].
- An accelerometer (Actigraph GT3X+; ActiGraph, Pensacola, FL, USA) will be used to measure levels of daily physical activity and general health [48–51].
- The Swedish National Board of Health and Welfare’s indicator questions about exercise and training habits [52] will be used to assess physical function.

Cognitive function:

- Computerized cognitive tests such as the AX-Continuous Performance Test (AX-CPT) and the Go/No-Go motor response inhibition test will be used to test mental function, and the International Affective Picture Series (IAPS) to test emotion regulation [53–55].
- An MRI examination with structural and functional brain imaging at rest (rest fMRI) and during brain activation, with cognitive (AX-CPT), motor (Go/NoGo) and affective (IAPS) paradigms [56–58], will be performed in 25 participants from each group.

Everyday functioning:

- The COPM [33, 34], a person-centred instrument that, during an interview, identifies, validates and prioritizes difficulties that negatively affect the person’s performance of activities in daily life, forms the basis for setting occupational therapy intervention goals. The person identifies performance problems and rates them on a scale of 1–10, where 1 = unable to perform at all and 10 = able to perform extremely well. After that, up to five difficulties are prioritized that the intervention should be aimed at. In the case of pre- and post-measurement, performance and satisfaction can be assessed separately. The COPM has been used in people of different ages, diagnoses and backgrounds. Reliability and validity as well as sensitivity to change are good [33, 34].
- The Assessment of Time Management Skills, Swedish version (ATMS-S) [58], will be used to assess time management. The ATMS-S is a self-assessment form consisting of 27 items divided into three subscales: time management skills (eleven items), organization and planning (eleven items), and regulation of emotions in relation to time (five items). The ATMS-S has been reported to have good psychometric properties [59].
- The Satisfaction with Daily Occupations-Occupational Balance (SDO-OB) [57] scale. This interview instrument contains 13 items that measure a person’s activity level and satisfaction with their daily activities as well as their perceived activity balance in the areas of: work/study (three items), leisure (three items), housework (four items), and self-care chores (three items). The SDO-OB scale has been developed in Sweden for people with mental disorders and exhibits good psychometric properties regarding internal consistency, validity and test-retest reliability [57].
- The Adult ADHD Quality of Life questionnaire (AAQoL) [56]. This is a self-assessment form containing 29 items that measure everyday functioning and QoL in areas that research has shown to have an impact on ADHD specifically. The form has four subscales: life productivity (eleven items), psychological health (six items), QoL (seven items), and relationships (five items). The AAQoL has been shown to have good psychometric properties [56, 60].
- The General Self-Efficacy ten-item Scale (GSES-10) [61]. This is a self-assessment form with ten items that are scored on a 4-point rating scale from “completely disagree” to “totally agree”. The GSES-10 measures confidence in one’s own ability to handle stressful situations. The instrument is used internationally and has shown good internal reliability; in this study, the Swedish version of the GSES-10 will be used [61].
- The Self-Efficacy for Exercise (SEE) scale [62]. This instrument will be used to measure self-efficacy barriers to exercise in the study population. The SEE-S is a 13-item instrument that focuses on self-efficacy expectations related to the ability to continue exercising in the face of barriers to exercise. There is evidence for reliability and validity of the SEE scale [62].
- Insomnia Severity Index (ISI) scale [63]. This is a brief self-report instrument consisting of seven items measuring the person’s perception of insomnia. Each item is rated on a 0–4 scale and the total score ranges from 0 to 28. A higher score suggests more severe insomnia. The ISI scale has been reported to have good reliability and validity [63].

Cost estimates for the health economic evaluation:

- Health care consumption and productivity loss (sick leave) will be estimated using data from administrative systems and/or medical records in health care. Costs will be estimated by multiplying frequencies by national Swedish tariffs and market prices. Total costs for each group will be aggregated over the trial period.
- Intervention cost will be estimated based on information provided by the persons responsible for the intervention.

### **Participants’ experiences of participation**

The experience of participating in the START intervention will be investigated using a qualitative approach where approximately 15 participants from each of the two intervention groups will be interviewed about their experience of participating in the intervention. The interviews will be conducted on one occasion, within 1 month after completion of the intervention. A study-specific interview guide will be used, covering experiences of the intervention in terms of content and its potential benefits or disadvantages. The interviews will be recorded and transcribed verbatim. Qualitative content analysis [64] will be employed. The data collection will take place at a venue of each participant’s choice and it is estimated that it will take 40–60 min.

### Analysis

#### Analysis of quantitative data

Descriptive analysis will be performed and the results will be reported using means ± standard deviations and ranges for continuous variables and numbers and percentages for categorical variables. Data will be analysed with a modified intention-to-treat (ITT) method and all patients involved in at least one data collection will be included in the analysis. Missing values will be imputed with multiple imputation method. Per-protocol (PP) analysis will also be performed where relevant. Analysis of variance (ANOVA) will be performed to compare means of the groups. The differences in baseline characteristics and post-intervention changes between the groups will be tested using *t*-test, Mann-Whitney U test, or Chi-squared test, whichever is appropriate. All analyses will be performed in the statistical software SPSS version 25.0 (IBM Corp., Armonk, NY, US).

#### Functional magnetic resonance imaging

A 3.0T MRI system (Signa Premier; GE HealthCare, Waukesha, WI, USA) will be used for structural and fMRI investigations. Data will be preprocessed with slicing-time correction, motion correction, smoothing and registration to a standard template. First-level analysis of the different paradigms (cognitive control, response inhibition and emotion regulation tasks) will be performed using a general linear model (GLM) with task-specific regressors.

#### Analysis of qualitative data

The semi-structured interviews will be analysed using qualitative content analysis. The focus in qualitative content analysis is on variations in the person’s experiences and presenting the results in categories and/or themes [64]. Initially the analysis process will involve getting to know the material, by reading and listening. Thereafter, codes will be generated and categories identified. The coding process will be discussed until agreement is reached between the authors; the codes will then be grouped into subcategories and categories that reflect the core message of the interviews. Thereafter, the subcategories will be abstracted into generic and main categories and the main theme. The data will be analysed using the Qualitative Data Analyses Software (QDAS) in NVivo (QSR International, Inc., Cambridge, MA, USA).

#### Health economic evaluation

A within-trial (3 months) and a long-term (1 year) cost–utility analysis will be performed from both health care and societal perspectives. The base case will consider health care costs and productivity loss. Gained QALYs will be used to measure effects. Cost-effectiveness ratios will be based on changes in QALYs and net costs of the intervention groups versus those in the control group. Results will be presented as an incremental cost-effectiveness ratio (ICER) expressed as (Ca – Cb)/(Ea – Eb). The uncertainty around the cost and effect estimates will be expressed in sensitivity analyses.

#### Monitoring harms

The purpose of monitoring is to maximize benefit and minimize harm. Patients will be asked to report any experienced adverse events from participating in the intervention. Any events that occur as a result of the activities of the study trial will be registered by the principal investigator (MM). Patients who experience an adverse event will be referred to health personnel for medical assessment. The research group has medical knowledge and knowledge of availablesupport.

#### Data storage

The S-T electronic data capture platform will be used. The S-T is a secure system and the content is tailored for this trial with web-based forms for each data collection point to facilitate, store and monitor outcomes. The platform is provided by RÖL and the security of the system is based on the rules and regulations concerning sensitive data. To log in as a researcher to all levels of the system, two-step verification is needed. For the participants, one-step verification is needed to register their answers. All communication with the database is encrypted and back-up is done regularly to secure data. Moreover, there is automatic marking of the event when data are missing or entered inaccurately.

It will be possible to efficiently follow each participant throughout the trial, and the data will be organized by unit. The system will therefore give a clear visual overview of each participant’s status. The use of the S-T fulfils the requirements of the European General Data Protection Regulation (GDPR). Only parts of the research group will have access to the S-T. For a researcher to gain access to the system, the principal investigator (MM) will have to approve this action, and will also have to determine what role the researcher who will be gaining access will have (e.g. entering or viewing data).

Directly after inclusion, participants will be registered in the S-T with their participant code and randomized to an intervention allocation. Raw data from physical data collection will be collected on paper forms, one bundle for each data collection point, containing all measures applicable to the specific occasion. The forms in the bundles will be in their original formats and will not have been changed by the research group. Once the data collection is completed and all raw data have been entered into the S-T, the data will be exported to statistical software for further analysis.

#### Ethics

Participants will receive oral and written information and informed consent will be obtained prior to inclusion. Since people with ADHD have a tendency to act impulsively, willingness to participate in the study will be assessed continuously. The risks of participating in the study are assessed as small. There will be a risk of feeling pain and sustaining injury related to exercise; however, this will be minimized by the fact that a physiotherapist will be present. Also, the programme is structured in such a way as to prevent overexertion and injury. Where participants might experience mental discomfort, support to deal with thoughts and feelings that arise will be available throughout the study. Ethical aspects that will be considered are challenges to personal integrity, confidentiality within the group and handling of negative reactions during assessment or treatment. All data will be anonymized before its use.

Ethical approval has been obtained from the Swedish Ethical Review Authority (EPM) (2020–05641; 2021–02743) and the study will be conducted in accordance with the Helsinki Declaration [65].

#### Approval and registration

The study has been approved by the Swedish Ethical Review Authority (No. 2020–05641 and 2021–02743 and 2022-03826-02), and is registered with ClinicalTrials.gov (NCT05049239). Any protocol modifications will be communicated to relevant parties.

## Discussion

This article presents a planned RCT to investigate whether a 12-week structured MEP with or without a cognitive intervention including cognitive skills training has an effect on core symptoms of ADHD in adults, as well as on physical, cognitive and mental function and everyday functioning compared with TAU.

The goal of the START intervention is to create evidence for a multimodal, non-pharmacological treatment for adults living with ADHD. Clinical studies in general face several challenges, such as the quality of the outcome measures used, as well as adherence to the intervention, and data collection [66]. In the planned RCT, adherence to the protocol and data collection may be especially challenging because of the symptomatology of adults with ADHD, such as difficulties in managing their time and planning and structuring their everyday life. To maximize adherence to the study protocol, different options will be suggested if participants miss a planned session. A minimum participation level of 50% will be set for the intervention group and participants missing a session will be offered an intervention session on another day of the same week or to catch up the planned exercise session at home and monitor and report their level of activity [26].

The START intervention may contribute to symptom reduction, a healthier and more active life, increased QoL, body experiences during exercise and better everyday functioning. Because of the complexity of the potential outcomes, many different measurements are needed to evaluate progress.

A general challenge in clinical studies is not to have an overwhelming data collection. In the planned RCT the rigorous data collection may be seen as challenging. On the other hand, a well-designed data collection strategy with different types of data and data collection options can be seen as a strength. The data collection with recurrent physical meetings in well-known facilities can ensure successful collection of data, and the collectors can catch up on missing data. Data-driven collections are easily performed on the one hand, but, as seen in our pilot study, they can also be a challenge for some participants [26]. Self-administration in data collections including the level of daily physical activity using an accelerometer as well as an HR monitor may also be a challenge. However, in our pilot study both self-administration and data-driven reporting of daily physical activity using an accelerometer and HR monitor were high, as was adherence to data collection stipulations.

In the planned RCT, participants randomized to the control group will be offered physiotherapy-supported training after the 12-week intervention period. This has both advantages and disadvantages. The positive effects of physical activity are well known and therefore it is considered unethical to not allow physical activity until the long-term follow-up has been completed. In our pilot study very few participants from the control group started physiotherapy-supported training after the 12-week period had ended. However, in the planned RCT study, participants in the control group who will start the physiotherapy-led intervention will not be included in the long-term follow-up and will be considered internal dropouts.

Another weakness of the planned RCT study is that the participants cannot be blinded to the treatment allocation regardless of which group they are in. This may create a conscious or unconscious expectation bias, which was also seen in the pilot study [26]. This is a major and insurmountable problem in the design, which is often seen in training intervention studies [66]. Another challenge is that participants may, during clinician-rated assessments, spontaneously and inadvertently mention the type of intervention they have received.

To gain knowledge of the participants’ own perspectives of the intervention and its eventual benefits, a qualitative study will be performed as part of the planned RCT study. To perform qualitative studies alongside RCT studies is recommended in complex health interventions such as START [67] as it can deepen the understanding of the other outcomes as well as add new research questions.

The potential personal gains from participating in the intervention, alongside the hypothesized health effects and increases in activity, include an easy-to-adopt new lifestyle that can, if implemented regularly, have several positive physical and mental effects. We therefore hope that the knowledge gained from this study will contribute to developing effective long-term strategies to reduce core symptoms and improve QoL for adult patients with ADHD.

## Data Availability

Not applicable.

## Declarations

### Trial status

Recruitment started in February 2021. Currently recruiting (50 out of 120 as of 9 June 2023); completion planned for June 2024.

## Abbreviations

AAQoL: Adult ADHD Quality of Life questionnaire
ADHD: attention deficit hyperactivity disorder
ANOVA: analysis of variance
ASRS-v.v1: Adult ADHD Self-Report Scale
ATMS-S: Assessment of Time Management Skills, Swedish version
AX-CPT: AX-Continuous Performance Test
BAS MQ-E: Body Awareness Scale-Movement Quality and Experience
BMI: body mass index
CGI-I: Clinical Global Impression-Improvement
CGI-S: Clinical Global Impression-Severity
COPM: Canadian Occupational Performance Measure
CS: central stimulant
DSM-IV-TR: Diagnostic and Statistical Manual of Mental Disorders, Text Revision, 4th edition
EB: Ekblom-Bak (exercise test)
EPM: Swedish Ethical Review Authority
EQ-5D-5 L: European Quality of Life Dimensions 5-level version
fMRI: functional magnetic resonance imaging
GDPR: General Data Protection Regulation
GLM: general linear model
GSES-10: General Self-Efficacy ten-item Scale
HR: heart rate
IAPS: International Affective Picture Series
ICER: incremental cost-effectiveness ratio
ICMJE: International Committee of Medical Journal Editors
ISI: Insomnia Severity Index
ITT: intention-to-treat (method)
MADRS-S: Montgomery-Asberg Depression Rating Scale, Self-Assessment
MEP: mixed exercise programme
MEP-C: mixed exercise programme-cognitive intervention
MRI: magnetic resonance imaging
PGI-I: Patient-rated Global Impression-Improvement scale
PP: per-protocol (analysis)
QALY: quality-adjusted life year
QDAS: Qualitative Data Analyses Software
QoL: quality of life
RCT: randomized controlled trial
RÖL: acronym for “Region Örebro County”
SDO-OB: Satisfaction with Daily Occupations-Occupational Balance
SEE: Self-Efficacy for Exercise (scale)
S-T: Smart-Trial
START: acronym for “*Stöd i Aktivitet, Rörelse och Träning*” (Support in activity, movement and exercise)
TAU: treatment as usual
VO_2_max: maximal oxygen uptake
VPN: virtual private network
WHO: World Health Organization
